# Military Graveyards and Epitaphs in India—III

**Published:** 1889-03-16

**Authors:** 


### MILITARY GRAVEYARDS AND EPITAPHS IN INDIA.—III.

BY AN OLD INDIAN.

(*Continued from page 368.*)

FOLLOWING previous examples, the prostrate partner of a blind old lady pours forth his soul in the following impassioned interrogatory to all whom it may concern—

"Was ever anything e'er so rare
E'en life itself half so dear
To mortal beings in this orb of light
As those blessed members the eyes sight?"

Bad as these are, there are others worse, and their incongruity and want of point are by no means their most objectionable features. It must be allowed, however, that these are amply atoned for and more than counterbalanced by the propriety and taste of some epitaphs that are to be found in their vicinity or elsewhere, and the inscriptions which represent the greatest of the Lawrences as "trying to do his duty" in the grassy end of the Residency at Lucknow; which commemorates the fall of General Neil in Huzrutgunge; and that which briefly describes the military career and death by cholera of Havelock in the Alumbagh, within sight of the object of all his struggles, are worthy of universal imitation. The same applies with some slight reserve to those over the graves of Nicholson near the Cashmere gate, Delhi, of the officers who fell during 1857, or who died previously and now repose in the old cantonment cemetery of that station near the Nujjufghur Canal. The memorial tablets with which the walls of St. James's Church in that city are so copiously studded are happily free from exaggeration, ill-feeling, and vindictiveness.

We are particularly pleased with one representing a poor fellow, a captain in the old Bengal artillery, as "lying in a soldier's grave dug on the field of battle in the hour of victory," which is to be found in the little whitewashed enclosure, near the present civil lines and on the very spot that witnessed the rout of the Sikh army, dedicated to the memory of those who fell at Goozerat. The memorial at Dum Dum to the officers and others of the engineer corps who died in 1857 is also appropriate; and the slabs that cover the remains of Colonel Pennnycuick and his son at Chillianwallah, still retain a certain amount of distinctness in the midst of a jungle. They contrast significantly with those of the "numerous dead" deposited hard by, and, flanked by the tablets of others equally brave though less known, are calculated to evoke feelings of compassion and regret for the fate of those whom their own too venturous ardour, the stratagem of a brave though less generous enemy, and the behest of a gallant but over-confident chief, consigned to inglorious slaughter.

And while these latter scarcely call for modification or change, it will, we think, be readily conceded that the former are but too much in need of amendment. Yet seeing the jealousy with which our laws watch over the liberty of the subject, and the objection our people have to anything savouring of officiousness or interference, we do not exactly see how this can be brought about. It is not easy to prescribe limits for the promptings of grief, and brevity in composition is not a favourite with the masses. Still something might be done in this direction. We do not want elaborate productions. It is not likely that any need will arise for such forcible language as that dictated by Johnson for the grave of Admiral Byng. The piteous wailing of Mr. Carlyle over his wife is not likely to prove attractive enough for imitation in India; and there is no poet's corner or poet of sufficient calibre to deserve the pithy inscription devoted to rare old Ben in Westminster Abbey."

The graveyards visited since this was written, early in 1867, include those at Rawul-Pindee, Nowshera, Peshawur, and Hoti-Murdan, and later still those at Abbottabad, Serinugger, and Murree. A few words at most will suffice for the majority of these. Of the cemetery at Rawul-Pindee, which contains the remains of the late Bishop Milman, nothing need be said, inasmuch as that it is a comparatively recent enclosure over which the local chaplains have exercised a rigid supervision, and from which they appear to have excluded all unseemly or irrelevant commemorations. A somewhat similar description will apply to the God's acre at Nowshera, with this difference, that it contains the bodies of some of the officers and men who fell at the Umbeyla Pass. The facts only, however, connected with these are recorded, and the following is the only inscription of interest noticed in it. The slab containing it was erected by his beloved and disconsolate widow to the memory of one Samuel Clement Parkhouse, a sub-collector in the Commissariat or Public Works Department, and is dated March 14th, 1869:—

His spirit in a robe of white
Has left us for awhile,
And winged its flight to God's right hand
And left a wife and child.
The prayer is done, the volley's fired,
And covered is the grave,
And here his body lies in peace
From "dust to dust"—it's gave.

Not far from the first-named place, on the Grand Trunk road, and on a rising ground that is dignified on the spot with the name of a pass, stands the obelisk that was erected, conjointly by natives and Europeans, to the memory of the celebrated John Nicholson. This officer, who was the friend of Edwards and of Lawrence, and who ruled the wild hillmen of Huzara with such wisdom and firmness as to assure peace among peoples who were quite unfamiliar with that phrase—while it ensured for himself the unparalleled honour of being worshipped during his lifetime as a demi-god—fell, as every-one knows, within the walls of Delhi, and his name is a "household word" to the readers of Kaye and Malleson. So well known, indeed, is he, that I need say no more about him, and were any further particulars requir ed, they are sup plied by the tablet there referred to:—

This Column Erected By Friends
British & Native
To the Memory of
Brigadier General John Nicholson C.B.
Who after taking a hero's part in 4 great wars for the defence of British India,
Cabul—1840
1 Seikh War...1845
2 Seikh War—1848
Sepoy Mutiny—1857
And being as renowned for his Civil rule
in the Punjab, as for his share in its
Conquest; fell mortally wounded
On 14th Sept. in leading to victory the
Main Column of assault, at the
Great seige of Delhi, and died 23rd
Sept 1857—aged 34.
Mourned by the two races with an equal grief.

One of the first objects that attracts the traveller's attention on his arrival at the great military station of Peshawur is the monument on the Mall, opposite the Artillery Hospital, that was erected by his friends to the memory of Colonel Mackeson, stabbed to the heart by a fanatical Khyberee, against whom some legal process had been recently executed, as he stood up in his kutcherry or office to receive a petition from him. This brave man's untimely fate has ever commanded sympathy and regret. The assassin was fortunately seized—though with great difficulty and danger—as he rushed down the declivity that led from the scene of his exploit towards the city, by a rifleman who happened to be passing at the time, and was summarily executed soon afterwards, *pour décourager les autres*. The monument itself supplies, though somewhat effusively, as I have often thought on the spot, such further particulars of the life and death of this gentleman as the reader may care to possess, and I am not aware that these have ever yet appeared in print:—

Here lies the body
of
Frederick Mackeson
Lieutenant-Colonel in the Bengal army Companion of the Bath
And Commissoner of Peshawur
Who was born Sept 2nd 1807
And died Sept 16th 1853

He was the beau-ideal of a soldier: Cool to Conceive, brave to direct, and strong to do: The Indian army was proud of his noble presence in its ranks: Not without Cause. On the dark page of the Affghan war the name of "Mackeson" shines brightly out: The Frontier was his post & the future was his field: The defiles of the Khyber & the peaks of the Black Mountain alike witness his exploits. Death still found him in the front: Unconquered enemies felt safer when he fell: His own Government thus mourned the fall:

"The repute of Col. Mackeson as a soldier is known to & honoured by all: His value as a political servant of the State is known to none better than the G.-General himself who in a difficult and eventful time had cause to mark his great ability and the admirable prudence, discretion and temper which added tenfold value to the high soldierly qualities of his public character.

"The loss of Col. Mackeson would have dimmed a victory." To lose him thus by the hand of a foul assassin is a misfortune of the heaviest gloom for the Goverment which counted him among its bravest and its best:

General orders of the
Marquis Dalhousie Gov. Gen. of India
3rd Oct 1853
This Monument was erected by his friends—

☞ Not far from this, in the same station—though whether in the cemetery or within the church I cannot just now remember—is a very similar, though somewhat less eulogistic, inscription. This too records the death, by the hand of an assassin, near the Cabul gate of Peshawur of Major Adams, and the interval between the two events (1853 and 1865) shows the intensity and persistence of the hate with which our race is regarded in these regions in much stronger colours than I could possibly supply. Poor young Ommaney's death, under like instigation, hard by at Hote-Murdan, occurred several years later still, and the recent attack on Captain Wintle would seem to imply that the old fire is only smouldering in that turbulent quarter. Whether it will burst into flame again remains to be seen, and meanwhile all will condole with the friends of him over whose mangled corse the following words were emblazoned:—

The Grave of
Robert Roy Adams
Major in H.M.'s Indian Staff Corps
And
Deputy Commissioner in the Punjab
Called to Peshawur as an Officer
Of rare Capacity for a frontier
Wise, just and Courageous
In all things faithful,
He came only to die at his post
Struck down by the hand of an assassin,
on the 22nd of January 1865
Aged 43.

Blessed are the dead that die in the Lord.—Rev. 14th, 13 v. For if we believe that Jesus died and rose again, even so them also which sleep in Jesus will God bring with him.—1 Thess. 4th, 14 v.

As to the two teeming* repositories that belong to the station itself, which I have often visited, as well with as without the usual accompaniments, they are situated between the cantonments and the Khyber hills, near the principal parade-grounds. They are known respectively as the Right Infantry and the Left Infantry Cemeteries, and they contain several interesting, though not many striking, epitaphs. There is nothing grotesque or irreverent in either, and the grave of Colonel Macdonald—who, like those others mentioned above, fell a victim to frontier fanaticism a few years ago—had not been covered in, *i.e.*, had not been made "pucka," as the phrase is, when I left that place.

On looking over the monuments in the first-named cemetery, I was particularly struck with the inscriptions I saw on the graves of some of the missionaries who have succumbed at that station. One of these, written in Hindustani, records the fact that the Rev. Isidore Lowenthal, a member of the American Presbyterian Mission, was accidentally (!) shot by his chowkedar on the morning of the 27th of April, 1864. This gentleman had just translated the New Testament into Pusthu, which fact may have possibly influenced his Mohammedan servant, and the following, copied from the stone that covers the remains of the Rev. Roger Edmund Clarke, B.A. (who died at the early age of 28), is very expressive:—

In great Joy and Peace
Trusting in Jesus
And Thankful to the Last
That He had been
A Missionary

As the 79th, the 42nd, and the 93rd Highlanders lost many men by cholera and otherwise in this station, so are there many Scotch names in its cemeteries, and a curious square block of solidified earth (chinam), that is surmounted by a short triangular spire, appropriately commemorates the names of the twenty sergeants of the first-named corps who died during its stay in India. There is a somewhat similar though less pretentious memorial-mound of the deaths that occurred in the 42nd in the same enclosure, and such Scriptural quotations as the following: "For he that is dead is freed from sin," "The Lord gave, and the Lord hath taken away," "He cometh forth like a flower, and is cut down," occur frequently. So also do expressions of hope or pious expectancy as to the future, and I have, I fancy, met the two following more than once on different tombs in this station. Though more suggestive of the religious fervour of the survivors than of any poetical inspiration, yet they are not inappropriate, and they are, I believe, not unfrequently found over the graves of children nearer home. The first was written about an infant, while the second indicated the last resting-place of a girl of two, and I introduce them here, as they are, I think, favourites everywhere in this sphere of life:—

Ere Sin could blight or sorrow fade
  Death came with friendly care;
The opening bud to heaven conveyed,
  And bade it blossom there.

God forbade her longer stay,
  God recalled the precious loan;
God hath taken her away
  From our bosoms to His own.
Surely what He wills is best,
Happy in His will we rest.

*(To be continued.)*

* I have myself seen as many as eighteen bodies interred in one of these enclosures from one regiment one evening, during a visitation of cholera in this station.

### A CONCERT AT NOTTINGHAM.

On the evening of Shrove Tuesday a most delightful musical entertainment was given to the patients of the Nottingham General Hospital. Through the kindness of Herr Ellenberger (who has done so much towards promoting the appreciation of good music in Nottingham) and his colleagues of the Chamber concerts, a most enjoyable programme was gone through. The concert commenced with Beethoven's string trio (serenade, Op. 8), performed by Herr Ellenberger (violin), Miss Tarbotten (viola), and Mr. E. Thorpe ('cello), which was listened to with rapt attention and heartily applauded. This was followed by songs, contributed by Mr. W. F. Bromley and others, after which "God Save the Queen" was sung, the patients joining in with great heartiness. The evening proved a thorough success, and the heartiest thanks of the institution are due to the *artistes* through whose kindness such a treat was provided. The concert was held in No. 7, one of the beautiful wards of the new wing.

A reaction from the laicigation of French hospitals may be inferred from the recent resolution, noted in a French medical journal, to establish a Catholic Faculty of Medicine at Lyons. A subscription list has been opened, and the first list of sums promised amounts to 400,000 francs (£16,000).

Chloral has a characteristic probably unique among sedatives, namely, that its action is powerful in proportion to the development of the brain. In idiots, a larger dose is required to produce effect than in those whose brains are fully developed, and highly-intellectual patients are peculiarly sensitive to its properties. Perhaps this latter fact explains the occasional cases of fatal poisoning from comparatively small doses of chloral given to patients of sensitive character.

